# Blood dendritic cells: “canary in the coal mine” to predict chronic inflammatory disease?

**DOI:** 10.3389/fmicb.2014.00006

**Published:** 2014-01-27

**Authors:** Brodie Miles, Khaled A. Abdel-Ghaffar, Ahmed Y. Gamal, Babak Baban, Christopher W. Cutler

**Affiliations:** ^1^Department of Periodontics, College of Dental Medicine, Georgia Regents UniversityAugusta, GA, USA; ^2^Ain Shams UniversityCairo, Egypt; ^3^Department of Oral Biology, Georgia Regents UniversityAugusta, GA, USA

**Keywords:** dendritic cells, inflammation, chronic infection, innate immunity, homeostasis

## Abstract

The majority of risk factors for chronic inflammatory diseases are unknown. This makes personalized medicine for assessment, prognosis, and choice of therapy very difficult. It is becoming increasingly clear, however, that low-grade subclinical infections may be an underlying cause of many chronic inflammatory diseases and thus may contribute to secondary outcomes (e.g., cancer). Many diseases are now categorized as inflammatory-mediated diseases that stem from a dysregulation in host immunity. There is a growing need to study the links between low-grade infections, the immune responses they elicit, and how this impacts overall health. One such link explored in detail here is the extreme sensitivity of myeloid dendritic cells (mDCs) in peripheral blood to chronic low-grade infections and the role that these mDCs play in arbitrating the resulting immune responses. We find that emerging evidence supports a role for pathogen-induced mDCs in chronic inflammation leading to increased risk of secondary clinical disease. The mDCs that are elevated in the blood as a result of low-grade bacteremia often do not trigger a productive immune response, but can disseminate the pathogen throughout the host. This aberrant trafficking of mDCs can accelerate systemic inflammatory disease progression. Conversely, restoration of dendritic cell homeostasis may aid in pathogen elimination and minimize dissemination. Thus it would seem prudent when assessing chronic inflammatory disease risk to consider blood mDC numbers, and the microbial content (microbiome) and activation state of these mDCs. These may provide important clues (“the canary in the coal mine”) of high inflammatory disease risk. This will facilitate development of novel immunotherapies to eliminate such smoldering infections in atherosclerosis, cancer, rheumatoid arthritis, and pre-eclampsia.

## INTRODUCTION TO BLOOD DENDRITIC CELLS

Tissue dendritic cells (DCs) are the peripheral sentinels of the human immune system (Banchereau and Steinman, 1998). As such, they play a seminal role in alerting the immune system so that a vigorous immune response can be mounted against a pathogen. This is balanced against the need of DCs to mitigate inappropriate responses toward commensal microbes or self-antigens (Banchereau et al., 2000). Circulating blood DCs are unique from tissue DCs in that they lack dendrites and do not express maturation markers such as CD83 (Ziegler-Heitbrock et al., 2010). They are identified by lack of Lin- (lineage) specific markers such as CD3, CD14, CD19, CD56, and glycophorin A and can be separated based on phenotype and function into three types: plasmacytoid DCs (pDCs) and two types of conventional or myeloid DCs (mDCs; Dzionek et al., 2000; Ziegler-Heitbrock et al., 2010; Hemont et al., 2013). pDCs, derived from lymphoid progenitors, are identified by the expression of CD123, CD303, and CD304. pDCs strongly express Toll-like receptor (TLR)-7 and -9, producing type-I interferons (IFNs) in response to CpGs but not lipopolysaccharide (LPS; Colonna et al., 2004; Gilliet et al., 2008). pDCs predominantly recognize viral antigens and are natural IFN producers (Colonna et al., 2004; Gilliet et al., 2008). mDCs are highly phagocytic, antigen-processing DCs that recognize both bacterial and viral antigens (Steinman and Inaba, 1999; MacDonald et al., 2002). The two subsets of mDCs are CD1c^+^ (BDCA-1^+^) mDCs and rare CD141^+^ mDCs. CD1c^+^ mDCs express all TLR except TLR-9, while CD141^+^ mDCs express a more restricted pattern with high expression of TLR-3 and -10, expression of TLR-1, -2, -6, and -8, and lack of TLR-4, -5, -7, and -9. The major response of CD141(+) mDCs to TLR-3 ligand and their cytokine production pattern suggest a role for these cells in antiviral immunity (Hemont et al., 2013). For this review, we will focus on mDCs.

mDCs are generated from common myeloid progenitors and are crucial in recognizing various pathogens, migrating through the lymph stream to secondary lymphoid organs (SLOs; Antonysamy et al., 1999) where they stimulate adaptive immunity and generate distinct CD4 effector T helper responses (Sallusto et al., 1998; Steinman and Inaba, 1999). Due to their great migratory capabilities and weak bactericidal mechanisms, mDCs in blood may be particularly susceptible to exploitation by pathogens, which this review will discuss in some detail.

## TYPICAL PHYSIOLOGICAL DEVELOPMENT OF BLOOD DC

Dendritic cells predominantly function within the confines of the tissues, lymphatics, and SLOs (Antonysamy et al., 1999). Blood mDCs are rare and comprise a very small population of total circulating leukocytes; usually below 1% of the total numbers. Circulating progenitors are typically divided into conventional DCs, which have archetypal DC functions and phenotype, as well as pre-DCs. The pre-DCs can be further developed into DC subtypes: HLA-DR^+^CD11c^+^ or HLA-DR^+^CD123^+^ (Pulendran et al., 2000). While very low in abundance in blood, mDCs are widely distributed in the body and can drive immune activation or tolerance, depending on state of activation or maturation, as we discuss later in this review. Additionally, mDCs exhibit typically short lifespans, especially after activation, and need constant replenishing. The influence of fluctuation of blood mDC number on host immune responses and homeostasis is unclear. What is clear is that mDCs are relatively rare in the blood, and rapidly mobilize in response to factors such as the DC-poietin Flt-3 ligand, and thus may be essential to normal physiology (Jefford et al., 2003; Karsunky et al., 2003). The hematopoiesis and generation of mDCs is complicated by the numerous subtypes of mDCs as well as the differences in steady state or inflammatory development (Shortman and Naik, 2007). The mDC system is generally in a state of constant flux, but this is intensified during infection and inflammation.

## DIFFERENTIATION OF mDCs FROM MONOCYTES

Blood monocytes are progenitors of mDCs with the ability to differentiate into various myeloid lineage cell types (Zhou and Tedder, 1996; Auffray et al., 2009). *In vitro* blood monocytes can be induced to differentiate into immature monocyte-derived DCs (MoDCs), by adding granulocyte macrophage colony-stimulating factor (GM-CSF) and interleukin (IL)-4 (Xu et al., 1995; Kiertscher and Roth, 1996; Palucka et al., 1998). MoDCs display very similar phenotype and functions as typical blood mDCs (Chapuis et al., 1997; Leon and Ardavin, 2008). Due to the low abundance of blood mDCs, this culturing technique has been essential for elucidating the functions of DCs. MoDCs (León et al., 2005) have been used to study the role of various stress conditions such as graft vs. host rejection (Antonysamy et al., 1999; Lutz et al., 2000), TLR stimulation (Krutzik et al., 2005), and autoimmunity (Blanco et al., 2001) and cancer (Thurner et al., 1999; Kiertscher et al., 2000; Schuler-Thurner et al., 2002; Figdor et al., 2004).

Myeloid precursors such as monocytes can rapidly differentiate into distinct populations of mDCs not typically present during steady state conditions, when encountering microbial and inflammatory signals. These signals can elicit rapid and sustained elevations of mDCs; such as occur during chronic, low-grade infections. This is typically discussed in the context of infection of peripheral tissues, which results in influx of CCR2^+^ blood monocytes to the site, where inflammatory stimuli promote differentiation of monocytes into mDCs (Geissmann et al., 2003, 2010; Cheong et al., 2010). TLR stimulation of monocytes appears to be essential for rapid differentiation of two distinct populations of MoDCs: DC-SIGN^+^CD16^+^ and CD1b/c^+^DC-SIGN^-^ (Krutzik et al., 2005). Additionally, recent work by our group demonstrates that low grade intracellular infection of monocytes stimulates their rapid differentiation into CD1c^+^DC-SIGN^+^ MoDCs (Miles et al., 2013a). Depending on the signals, however, these increases do not always correlate with productive immune responses, as the resultant mDC pool can often be immuno-incompetent. Hence, an increase in mDCs during chronic infections may further exacerbate inflammatory diseases through faulty pathogen elimination and antigen presentation. Therefore, it becomes extremely important to quantitate and characterize the activation state of mDCs that are mobilized in the blood in response to low-grade infections. These observations might have potential in a clinical setting, as both a way to predict disease risk and as a targeted therapy approach.

## TRANSMIGRATION OF mDCs TO AND FROM TISSUES

Upon antigen acquisition, DCs undergo a maturation process characterized by downregulation of their phagocytic machinery and upregulation of their antigen presenting capacity. The costimulatory (and coinhibitory) molecules on DCs that alter antigen presentation by DCs are discussed below. The process of DC maturation occurs simultaneously with an upregulation of the chemokine receptor CCR7 (Forster et al., 2008), which directs mature DCs to different lymphoid compartments where a gradient of CCL19/21 is present (Randolph et al., 2008). The functions of specific chemokine receptors on DCs and their ligands have been previously reviewed (Mohit and Rafati, 2012) and will not be repeated here. The process of chemokine receptor modulation on blood DCs and their precursors drives inflammatory influx into tissues. Once in the tissues, DCs also possess the capability to reverse direction, cross the endothelial barrier, and redistribute into blood circulation (Muller and Randolph, 1999; Sozzani et al., 1999; Gordon and Taylor, 2005); however, direct evidence of this reverse transmigration phenomenon is scarce. Notably, CD16^+^ inflammatory monocytes have been shown to enter the circulation from tissues in one study (Randolph et al., 2002) and human MoDCs were able to reverse transmigrate through human umbilical vein endothelial cells *in vitro* (D’Amico et al., 1998). The endothelium plays a principal role as a barrier to inflammatory infiltration from the vasculature, but also facilitates DC transmigration particularly in response to inflammatory signals (Weis et al., 2002) and hypoxia (Schioppa et al., 2003; Mancino et al., 2008). During infection, it is hypothesized that transmigration contributes to the pathophysiological function called pathogen-trafficking, whereby DCs carry microbes from peripheral tissues into peripheral blood to distant sites, thus contributing to inflammatory diseases, such as cardiovascular disease (CVD; Ross, 1999; Niessner and Weyand, 2010; Zeituni et al., 2010a) and arthritis (Thomas et al., 1999; Wang et al., 2006).

## NORMAL mDC FUNCTION: TISSUE vs. BLOOD

The role of DCs in immunity can also be subdivided based on the predominant anatomical compartment in which they are found. Lymphoid resident DCs are non-migrating DCs that normally reside in the thymus and spleen, where they collect and present antigen (Kabashima et al., 2005). These include tissue resident DCs, namely Langerhans cells and interstitial DCs (intDC), which have relatively long lifespans. This allows them to survey their local environment for danger signals or apoptotic host debris. DCs actively clear away debris, take up and process antigens, or alternatively, promote host tolerance. However, nearly half of the DCs found in these tissues are not typical lymphoid resident DCs, but consist of the classical migratory DC subsets. The proper danger signals must be present to stimulate DC maturation that allows DCs to respond to chemokine gradients originating in peripheral lymph nodes (Luther et al., 2002). This tightly regulated system allows stratification of DC functions according to anatomic site, thereby mitigating peripheral antigen presentation, while favoring antigen-specific immune responses at SLOs (Dieu et al., 1998; Balázs et al., 2002). While lymphoid resident DCs are generally immature and actively take up antigen, the classical migrating DCs have already matured and lost antigen uptake capabilities by the time they reach the lymph tissues.

As stated earlier, the predominant functions of blood mDCs are poorly understood. Immature blood DCs and monocytes tend to have similar functions, but can drive a different scale of response when matured. Peripheral blood mDCs are differentially regulated by the cytokine milieu, which leads to alterations in surface expression of major histocompatibility complex class II (MHC-II) and accessory molecules (Kohrgruber et al., 1999). Hence, these circulating cells appear capable of processing antigen and stimulating adaptive immunity, but this can be manipulated by their environment (Thomas and Lipsky, 1994; Ito et al., 2001). It is widely speculated that they may migrate in and out of tissues to replenish tissue resident DCs (Ito et al., 1999; Varol et al., 2007; Merad and Manz, 2009), but they also appear to have an active role in clearance of bacteremia (Fanger et al., 1996; Balázs et al., 2002; Carrion et al., 2012).

## ACCESSORY MOLECULES: COSTIMULATORY AND CO-INHIBITORY

In addition to DC number and location, the expression of accessory molecules must be considered, as DCs must strike a balance between promoting immunity and immune tolerance. They are able to guide and direct the effector functions of T cells toward immunogenic CD4^+^ T helper cells and cytotoxic CD8^+^ T cells or toward tolerogenic regulatory T cells (Tregs; Bakdash et al., 2013; Hubo et al., 2013). This directorial function depends on three major signals that govern the cross talk between antigen-presenting DCs and responding T cells. Signal 1 consists of processed antigen peptide presented in the context of MHC complexes to the specific T cell receptors (TCRs). Signal 2 consists of upregulation of costimulatory/coinhibitory molecules for binding to their respective receptors on T cells. Understanding the components of costimulation/coinhibition mechanism is highly crucial for elucidation and interpretation of immune responses and is discussed further here (Bakdash et al., 2013). Signal 3 consists of the proper repertoire of secreted cytokines, providing the final directions to the emerging T cells.

The B7 family of costimulatory molecules (CD80/CD86) are the best defined, and perhaps, most biologically significant costimulatory molecules involved in T cell activation. Interaction between CD80 (B7.1) and CD86 (B7.2) on DCs (as well as on macrophages and B cells) and their binding partner CD28 on T cells (mainly on CD4^+^ T cells and some with less frequency on CD8^+^ T cells) initiates the B7 costimulatory pathway. This occurs through tyrosine phosphorylation and activation of the PI3K/AKT, which, along with TCR signaling, promotes IL-2 gene expression and cellular proliferation. This activation also occurs simultaneously with the upregulation of anti-apoptotic genes. The B7/CD28 costimulatory reaction not only initiates the activation and proliferation of effector T cells, but also negatively affects the tolerogenic capabilities of DCs and thereby reduces the induction and activation of Tregs. Interestingly, it is well demonstrated that activation of T cells through costimulatory molecules can be contained by a negative feedback controlling system. This involves polarization of CTLA-4 on activated T cells. CTLA-4 can compete with CD28 costimulatory molecule to bind B7 ligands on DCs with higher affinity, resulting in suppression of effector T cells and reduction in inflammatory responses (Bour-Jordan et al., 2011; Hubo et al., 2013).

The other major member of B7 costimulatory family is the ICOS (CD278)/ICOS-L (CD275) interaction. The ICOS/ICOS-L pathway is a crucial player in T cell-dependent antibody response to the antigens. Interestingly, some studies have shown co-inhibitory functions for the ICOS/ICOS-L pathway involving the production of anti-inflammatory cytokines, IL-10 and IL-4, as well as induction of Tregs. This assigns a novel potential immunotherapeutic role for ICOS/ICOS-L pathway in the treatment of autoimmune diseases (Bakdash et al., 2013; Hubo et al., 2013).

On the inhibitory side, the two ligands of programed death 1 (PD-1), PD-L1 and PD-L2, have emerged as crucial co-inhibitory molecules. PD-L1 and PD-L2 initiate pathways for immune tolerance and modulation of T cell responses. Interaction of PD-1 with PD-L1 and PD-L2 can effectively suppress T cell responses and lead to induction of Tregs. Stimulants, such as endotoxins (LPS) or other immunostimulatory signals like CD40, may result in PD-L1 signaling and immunosuppression. Unlike PD-L1, PD-L2 is exclusively expressed by antigen-presenting cells (APCs; e.g., dendritic cells, macrophages). Altogether, the PD-1 pathway is one of the major mechanisms responsible for the establishment of a tolerogenic microenvironment, which can play an important role as a therapeutic target in treatment of autoimmune and inflammatory diseases (Bour-Jordan et al., 2011; Riella et al., 2012; Bakdash et al., 2013; Hubo et al., 2013).

The CD40/CD40-L pathway is particularly influential in T cell differentiation and sensitization. CD40-L, a member of the tumor necrosis factor (TNF) superfamily, is presented on activated T cells. This pathway can function in a dichotomous fashion, promoting immunity or inducing tolerance, depending on the nature of the cytokines and other factors in the microenvironment. OX40-L, which is presented on APCs, has similar dual and complex function as CD40-L when reacts with CD40 (Hubo et al., 2013). Collectively, the function of costimulatory/co-inhibitory pathways on DCs is pivotal for immunity or tolerance.

## LINKS BETWEEN CHRONIC INFECTIONS AND mDC FLUCTUATIONS

Low-grade chronic infections are detrimental to host health as they routinely cause inflammatory flare-ups, leading to host tissue destruction. Implicit in this argument is that immune function is impaired in the process, allowing pathogen persistence. For productive immune responses, mDC must function to strongly stimulate and polarize the innate and adaptive branches of immunity. As mDCs are not efficient killers of microorganisms in their own right (Palucka and Banchereau, 2002; Geijtenbeek et al., 2003; Macpherson and Uhr, 2004), they may provide a protective niche for pathogens. One of the key events in many infections that have been linked with systemic illness is pathogen dissemination (Kelsall and Leon, 2005; Finlay and McFadden, 2006; Caws et al., 2008; Brandtzaeg, 2009; Hayashi et al., 2010). This is explored in **Table 1**. As a result of uptake and survival within mDCs, pathogens consequently gain access to a highly migratory “vehicle” for dissemination.

**Table 1:** Association of shifts in blood dendritic cell numbers with inflammatory/infectious diseases.

Disease (pathogen)	Immune function of mDCs during infection	Changes to mDC levels	Phenotype markers used	Pathogen dissemination	Reference
Chronic periodontitis (*P. gingivalis*, others)	Intracellular bacterial survival; lack of DC maturation and immune signaling, aberrant migration	Increased blood mDC	CD1c^+^, DC-SIGN^+^, CD19^-^	Yes	Carrion (Carrion et al., 2012), Cutler (Cutler and Jotwani, 2004; Cutler and Teng, 2007), Miles (Miles et al., 2013a,b)
HIV	Trans-infection of T cells, HMGB1 promotes dissemination and latency	Decreased blood mDC; increased shortly after ART	HLA-DR^+^, CD11c^+^, CD123^-^	Yes	Chehimi (Chehimi et al., 2002), Engering (Engering et al., 2002), Feldman (Feldman et al., 2001), Gougeon (Gougeon et al., 2012), Grassi (Grassi et al., 1999)
Gastrointestinal inflammation *(H. pylori*, others)	Increased IL-17 secretion and Treg stimulation, localized inflammation	Increased mucosal mDCs	HLA-DR^+^, CD11c^+^	Yes	Baumgart (Baumgart et al., 2005), Vuckovic (Vuckovic et al., 2001), Xavier (Xavier and Podolsky, 2007)
Pneumonia (*C. pneumonias*)	Matured, antigen presenting, Th-2-inducing immunity	Relatively unchanged	HLA-DR^+^, CD11C^+^	Yes	Ojcius (Ojcius et al., 1998), Wittkop (Wittkop et al., 2006), Bobryshev (Bobryshev et al., 2004)
Tuberculosis (*M. tuberculosis*)	Promote HIV trans-infection, systemic dissemination from granulomas in both acute and chronic stages, impaired Ag-presentation	n/a	HLA-DR^+^, CD11c^+^, CD123^-^	Yes	Chackerian (Chackerian et al., 2002), Gringhuis (Gringhuis et al., 2009), Lichtner (Lichtner et al., 2006), Tailleux (Tailleux et al., 2003), Wolf (Wolf et al., 2007)
Diabetes mellitus	Activation of autoreactive T cells, autoimmunity, low accessory molecules	Increased blood mDCs	CD11c^+^	n/a	Lee (Lee et al., 2011), Lo (Lo and Clare-Salzler, 2006), Surendar (Surendar et al., 2012)
Asthma and allergies	Promote inflammation, recruit mast cells, eosinophils, etc., phagocytose allergens	Increased blood and mucosal mDCs	HLA-DR^+^, CD11c^+^	n/a	Idzko (Idzko et al., 2007), Lambrecht (Lambrecht and Hammad, 2003), Parameswaran (Parameswaran et al., 2004), Spears (Spears et al., 2011), Upham (Upham et al., 2002)
Chronic hepatitis C virus (HCV)	Niche for viral replication, normal IFN-α production, Th-17 bias	Increased/decreased blood mDCs	HLA-DR^+^, CD11C^+^	Yes	Fang (Fang et al., 2013), Goutagny (Goutagny et al., 2003), Longman (Longman et al., 2005), Musilli (Musilli et al., 2011), Wertheimer (Wertheimer et al., 2004)
Langerhans cell histiocytosis (LCH)	DC recruitment via hematopoietins	Increased blood mDCs	HLA-DR^+^, CD11c^+^	Yes	Abla (Abla et al., 2010), Laman (Laman et al., 2003), Rolland (Rolland et al., 2005)

As shown in **Table 1**, there are common the themes in the ability of chronic and acute pathogens to cause alterations in relative mDC number and the ability to disseminate within mDCs. There can be a localized inflammatory response, commensurate with pathogen dissemination by non-stimulatory mDCs (i.e., immature mDCs), as seen with diseases such as chronic periodontitis (CP), gastritis, asthma, and tuberculosis.

## EXPANSION OF mDCs DURING CHRONIC PERIODONTITIS

A recent clinical study by our group (Carrion et al., 2012) showed that CD1c^+^DC-SIGN^+^ mDCs were expanded in the peripheral blood of subjects with CP. This rise in mDC numbers was expanded further when the CP subjects also had acute coronary syndrome, a form of CVD. This was not due to an overall increase in total peripheral blood mononuclear cells (PBMCs), nor was it due to a rise in DC-poietins that would otherwise account for the increased mDCs in circulation. The circulating mDCs were shown to carry the oral pathogen *Porphyromonas gingivalis*, along with a diverse microbiome. Moreover, DC-SIGN^+^ mDCs shown to contain *P. gingivalis* were identified within the coronary artery plaques *in situ*. As *P. gingivalis* targets DC-SIGN for entry into mDCs via its glycoprotein fimbriae, mfa-1 (Zeituni et al., 2009, 2010b), this was deemed to have particular significance to the pathophysiology of both CP and CVD. CP and CVD have been linked epidemiologically, but the mechanisms involved in this association are unclear (Dietrich et al., 2013). The results of our clinical study (reviewed in Zeituni et al., 2010a) and two follow-up studies indicate that the microbial carriage state of mDCs and their progenitors monocytes activates DC differentiation (Miles et al., 2013a) and promotes trafficking of these infected mDCs to sites of neovascularization (Miles et al., 2013b) such as diseased coronary arteries, thereby increasing the CVD risk associated with CP.

As with other diseases highlighted in **Table 1**, the elevated mDCs in CP lack proper accessory molecule expression for driving robust adaptive immune responses. Hence, it appears that while blood mDCs serve an important clearance function for bacteremia, when faced with a microbe that targets DC-SIGN or other immunoregulatory routes, this clearance may be detrimental to the host (Zeituni et al., 2009).

## CORRELATION OF MICROBIAL AND NON-MICROBIAL FACTORS WITH FLUCTUATIONS IN mDC NUMBERS

Both non-microbial and microbial stimuli can induce fluctuations in blood mDC numbers, ostensibly through different mechanisms. Inflammatory asthma is an example of a non-microbial disease that elicits elevations in certain peripheral blood DC subpopulations. This elevation in DCs is believed to be important in the development and maintenance of symptoms (Upham et al., 2002; Parameswaran et al., 2004; Spears et al., 2011). Moreover, the activation of DCs is an important mediator of continued inflammation in asthmatic patients (Lambrecht and Hammad, 2003; Idzko et al., 2007). Another non-microbial disease, Langerhans cell histiocytosis (LCH), results in increased circulating mDC levels commensurate with a rise in hematopoietic cytokines (Rolland et al., 2005). The cause of this rise in blood mDCs is unclear, but it has been speculated that this imbalance may replenish tissue DCs that have migrated to lymph nodes, or represents reverse transmigration of DCs into blood circulation (Laman et al., 2003). Prominent among the microbial stimuli is *M. tuberculosis*, a particularly effective pathogen by virtue of its ability to infect mDCs at a high rate and to obtund antigen presentation (Wolf et al., 2007). It also expands the circulating mDC pool with mDCs that lack IFN-α production necessary for effective immune function (Lichtner et al., 2006). The circulating mDCs associated with *M. tuberculosis* infection also drive pathogen dissemination prior to the development of a productive T cell response (Chackerian et al., 2002). Pneumonia, induced by pathogenic species such as *Chlamydia pneumoniae*, is associated with increased circulating mDCs that disseminate the pathogen to various sites in the host (Ojcius et al., 1998). The mDCs generated during *C. pneumoniae* infections in one report are functionally suppressive. The mDCs disseminate the pathogen and drive immunosuppressive T cell stimulation (Wittkop et al., 2006). Viruses (e.g., hepatitis C virus, HCV) can also infect mDCs and expand suppressive immune responses, typically through Th-17 response (Fang et al., 2013). HIV infection is known to cause immunosuppressive responses such as impaired effector T cell function, cell death, and Treg induction during progression to chronic disease. HIV tropic virus can infect mDCs and subsequently trans-infect T cells (Engering et al., 2002). The blood mDC responses in patients with inflammatory bowel disease (IBD) vary widely, from reports of no change in mDC numbers to elevated numbers. Regardless, the mDCs are typically activated and hyperstimulatory (Vuckovic et al., 2001; Baumgart et al., 2005, 2009; Xavier and Podolsky, 2007).

During acute infections, elevations in non-functional or immunosuppressive mDCs is a common occurrence. For example, infections with *P. falciparum*, dengue virus, or influenza virus typically trigger a rise in circulating blood mDCs which may favor pathogen dissemination and chronic inflammation (Aldridge et al., 2009; Cao et al., 2012; Ibitokou et al., 2012; Torres et al., 2013). During sepsis and shock, rapid elevations of mDCs occur through differentiation of precursors in blood (Faivre et al., 2007, 2012). Conflicting reports of sustained decreases in circulating mDCs in septic shock are also evident (Inoue et al., 2010; Grimaldi et al., 2011). Other conflicting reports are also evident. During HIV infection, mDCs numbers generally decrease in the blood circulation (Grassi et al., 1999; Feldman et al., 2001), but are increased in HIV patients after treatment with highly active antiretroviral therapy (HAART; Chehimi et al., 2002). In addition, the blood mDC levels during HCV infection and chronic liver disease are somewhat controversial, with both reports of decreases (Goutagny et al., 2003; Longman et al., 2005) or no changes (Wertheimer et al., 2004). As DC-SIGN is used as a ligand for HIV (Kwon et al., 2002; Gringhuis et al., 2009) and is one of many targets for HCV entry (Lozach et al., 2003), mDC ligation via DC-SIGN and the resulting intracellular routing could explain some irregularities of subsequent DC function.

## A ROLE FOR mDC FLUCTUATION AND INFLAMMATORY RESPONSE IN DIABETES

The importance of mDC function is also highlighted in autoimmune diabetes, in which mDC populations are increased, and drive activation of autoreactive T cells (Lo and Clare-Salzler, 2006). This supports our underlying common theme that diseases of microbial and non-microbial origin have in common a dysregulation in mDC homeostasis, resulting in dissemination of pathogens, allergens, and/or self-antigens. In each instance, the dysfunction in mDCs as a result favors pathogen/allergen persistence and prevents effective antigen-specific immunity and clearance.

Both autoimmune and type-II diabetes, widespread diseases that are tied very closely with immune function, have been linked with CP and mDC dysfunction. Circulating mDC populations are increased in both type-II diabetic patients and obese diabetic patients (Musilli et al., 2011; Chmelar et al., 2013). Several oral pathogens are elevated during non-insulin-dependent diabetes mellitus and may play a role in progression of both diabetes and periodontal disease severity (Yuan et al., 2001). The development of diabetes mellitus contributes to the exacerbation of periodontal disease and ligature-induced periodontal disease in turn has been linked to decreased insulin sensitivity in rats (Colombo et al., 2012). In contrast, diabetes in mice was found to be unchanged upon induction of experimental periodontitis by *P. gingivalis* *in vivo* (Li et al., 2013). Evidence is beginning to point toward a role for periodontal inflammation in development of type-II diabetes and that inflammatory mediators produced by monocyte/macrophages during immune responses to periodontitis may lead to insulin resistance (Nishimura et al., 2003). Hence, it is now believed that these diseases form a bidirectional relationship.

The use of animal models has also demonstrated that periodontal symptoms upon *P. gingivalis* infection are worsened with diabetes (Ensminger et al., 2008), but the mechanisms are unclear. Further, TLR-2 activation and signaling, which are pronounced during CP and which are crucial for mDC function against *P. gingivalis* (Asai et al., 2007; Kanaya et al., 2009), have been found to play a role in autoimmune diabetes progression (Kim et al., 2011; Lee et al., 2011). Levels of DC-poietins, such as GM-CSF, were observed to be elevated in diabetes patients, which correlated to increased numbers and activation state of mDCs in peripheral blood. Interestingly, normal levels of GM-CSF and mDCs were restored upon combination therapy with insulin and oral hypoglycemic agents (Surendar et al., 2012). This suggests that with proper diagnosis, blood mDC levels may be restored and symptoms of inflammatory autoimmune diabetes averted.

### DENDRITIC CELL FREQUENCY AS PREDICTIVE MEDICINE

The damage to the host caused by chronic diseases are often irreversible; thus the timing of risk assessment is crucial to prevention of adverse health effects. For example, the diagnosis of LCH can be missed or delayed until symptoms and systemic dysfunction became widespread (Abla et al., 2010). In IBD, circulating and tissue mDCs are highly active and display an easily detectable mature phenotype (Hart et al., 2005). Enumeration and characterization of accessory molecules on blood mDCs could provide a minimally invasive and accurate tool to diagnose disease risk and prevent late-stage symptoms of chronic inflammatory disease. Attempts to address the links between microbial organisms and systemic disease have been made recently through the use of large clinical trials. Specifically, patients with cardiovascular illness have been treated with systemic antibiotics to ascertain if elimination of bacteremic and disseminating pathogens could improve clinical outcomes. These treatments have been largely unsuccessful (Andraws et al., 2005; Gurfinkel and Lernoud, 2006), but it is unclear if these antibiotics effectively eliminate intracellular pathogens. Hence, continued improvements to assess mDC levels and intracellular antibiotic levels are necessary to fully assess patient outcomes.

In addition, the frequency and subtype of mDCs in blood may shed light on the development of autoimmune disease before symptoms become pronounced. Increased blood mDC differentiation occurs in autoimmune systemic lupus erythematosus (SLE) and could potentially be used to determine and limit the severity of autoreactive B and T lymphocyte function (Gill et al., 2002). Treatment of DCs with vitamin D receptor ligands has been found to inhibit their alloreactive T cell stimulatory capacity and enhances their Treg activation, which can resolve autoimmune disease (Griffin et al., 2001; Adorini, 2003; Adorini et al., 2004).

Recent work in our lab and others show that there may be a window of opportunity to assess blood mDC populations before the acceleration of systemic inflammatory diseases. It appears that the low-grade infections of chronic and acute nature highlighted here, elicit disruption of blood DC homeostasis. This disruption of DC homeostasis can contribute to the early or late progression of inflammatory disease. DC populations are not detected by typical complete blood counts in clinical laboratories. When they are, it is because severe manifestations of inflammatory disease are present. At this stage, the window of opportunity is lost, as mDCs have already infiltrated tissues, coinciding with a decrease of circulating mDCs. Therefore, monitoring of mDC levels and their activation state in blood, especially during chronic infection, should be further investigated as a predictive tool for additional disease and chronic inflammatory risk.

## PATHOGEN-DRIVEN DENDRITIC CELL EXPANSION AND DISSEMINATION

The oral mucosal infection CP is characterized by accumulation of a pathogenic biofilm on the tooth surface, which eventually leads to a high degree of tissue and bone loss caused by the host response (Casarin et al., 2010; Darveau, 2010). Certain microbial species, notably among them the anaerobic Gram-negative bacterium *P. gingivalis* (Byrne et al., 2009; Hajishengallis et al., 2012), produce destructive proteolytic enzymes and lead to spikes in inflammatory responses (Hajishengallis, 2009). Among these inflammatory responses is the infiltration and activation of mDCs (Cutler and Jotwani, 2004, 2006; Cutler and Teng, 2007). These mDCs express the C-type lectin and pattern recognition receptor (PRR) DC-SIGN, which is used as an invasin by a broad range of pathogenic organisms (Lozach et al., 2003; Tailleux et al., 2003; Gringhuis et al., 2009; Mesman et al., 2012). In the case of *P. gingivalis*, a fimbrial adhesin is expressed, the 67-kDa minor fimbriae (mfa-1), which is glycosylated and targets DC-SIGN for entry into mDCs (Zeituni et al., 2010b). This interaction has also been shown to skew mDC function away from maturation, leading to low accessory molecule expression and an immunosuppressive cytokine production (Zeituni et al., 2009).

## NON-CANONICAL PATHOGEN-DIFFERENTIATED DCs

**FIGURE 1:**
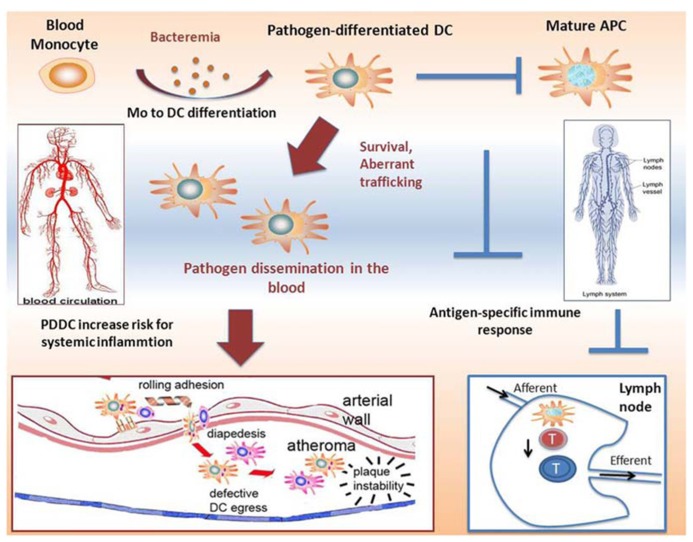
**Proposed mechanisms of induction of immunosuppressive pathogen-differentiated dendritic cells (PDDCs).** Low-grade bacteremia, such as in chronic periodontitis, stimulate monocyte to dendritic cell differentiation, resulting in immature non-canonical PDDCs. These long-lived PDDCs contain viable bacteria and do not home through the lymphatics to secondary lymphoid organs where they would be able to prime effector T cells. Rather, infected PDDCs are able to circulate through the blood and traffic to various inflammatory sites, such as atherosclerotic plaques. After migrating to these sites, they further the extent of local inflammation and adversely contribute to systemic illness.

More recent *in vitro* findings in our lab show that *P. gingivalis*, through its interaction with DC-SIGN, rapidly induces differentiation of MoDCs from monocyte progenitors. These DCs, which we termed pathogen-differentiated DCs (PDDCs), are CD1c^+^DC-SIGN^+^CD14^-^, but immature in phenotype and function unless forced to mature with inflammatory cocktail. It is believed that intracellular routing through DC-SIGN allows for pathogen survival in passive compartments that avoid lysosomal fusion and is a current line of investigation by our group. In addition, this interaction further dysregulates DC homeostasis by driving an aberrant chemokine receptor profile. As highlighted in **Figure 1**, this has implications for both pathogen dissemination and immune subversion. Bacteremia is frequent and transient during CP (Parahitiyawa et al., 2009; Pérez-Chaparro et al., 2009; Morozumi et al., 2010), which could drive sustained blood mDC increased through generation of non-canonical PDDCs. These PDDCs would provide a protective niche for the pathogen, obviating antigen processing and presentation required for effective immune response. Finally, these PDDCs display poor lymphoid-homing capabilities (Miles et al., 2013b) which prevents them from stimulating effector cell responses. Instead these PDDCs circulate through bloodstream and infiltrate distant sites, with the arterial wall depicted, which leads to localized inflammation and negatively effects systemic health.

## BLOOD mDCs AS CARRIERS OF INFECTION

Other pathogens can influence DC differentiation and activation, while occupying a protective niche within DCs to escape immune clearance. Mucosal pathogens HCV (Goutagny et al., 2003), *C. pneumoniae* (Wittkop et al., 2006), HIV (Gougeon et al., 2012), and *M. tuberculosis* (Chackerian et al., 2002), among others, survive and replicate within DCs. Interestingly, many of these pathogens are found to be disseminated to distant sites within the host and thought to have a role in inflammatory disease (Bobryshev et al., 2004). Evidence suggests that these mucosal pathogens are carried to distant sites within DCs, serving as vehicles of dissemination (Bobryshev, 2005; Bosio, 2010; Niessner and Weyand, 2010).

## POTENTIAL ROLE FOR NOVEL DC DIFFERENTIATION IN IMMUNOTHERAPY

Due to their high degree of plasticity, mobility, and ability when mature to stimulate a robust specific immune response, DC subsets are attractive targets for immunotherapy and vaccine development (Fong and Engleman, 2000; Ueno et al., 2010). Different DC subsets display different recognition receptors and produce different cytokine profiles, which can lead to fine tuning of specific desired immunological outcomes (Klechevsky et al., 2009). The use of DC vaccines also provides an opportunity to stimulate not only humoral immunity, but cellular immunity as well (Palucka et al., 2010). This is crucial to developing a vaccine currently lacking, for intracellular pathogens such as HIV, HCV, and tuberculosis. DC vaccines are also attractive as cancer therapies. The use of DCs can allow for specific immunity against subtle differences in host cells while preventing widespread inflammation and damage seen with chemotherapeutic agents. DC-based immunotherapy in clinical trials for cancer is a particularly exciting field (reviewed in Vacchelli et al., 2013). Various challenges in this regard include poor immunogenicity of target cells, insufficient function of transplanted immune cells, low immune stimulation by *in vitro* generated DCs or short lived nature of these DCs (Bronte et al., 1997; Wang et al., 1998; Rosenberg et al., 2004).

**FIGURE 2:**
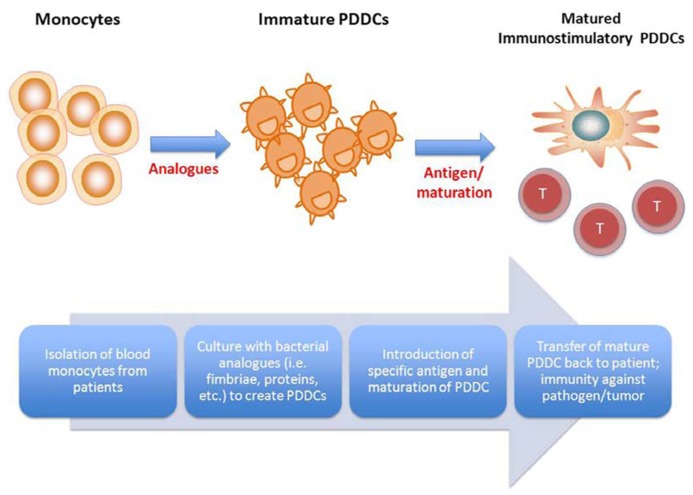
**Proposed therapeutic use of pathogen-differentiated dendritic cells (PDDCs).** *In lieu* of using traditional GM-CSF/IL-4-cultured monocyte-derived DCs for immunotherapy, monocytes are treated with bacterial analogs to induce iTreg- or Th-17-inducing PDDCs, and PDDCs loaded along with specific antigen, depending on the disease to be treated.

In **Figure 2**, we propose a flow diagram for the development of a DC vaccine that would take advantage of PDDCs and promote a tailored, specific immune outcome. PDDCs could be generated with various bacterial analogs, such as was shown with *P. gingivalis* mfa-1. These PDDCs are longer lived *in vitro* than conventional MoDCs and require additional maturation (Miles et al., 2013a). Large populations of PDDCs could be generated from abundant monocyte precursors that display the desired immunogenicity and T helper cell stimulating ability. These PDDCs can then be transferred back to patients and either drive antigen-specific immunity or prevent inflammation and autoimmunity through immune suppression. By using the various subsets of PDDCs, immune responses can be tailored to produce stimulatory or tolerogenic effector responses. Therefore, it is plausible that DC immunotherapy could potential target autoimmune diseases. Evidence already suggests that mDCs can trigger immunosuppressive responses to reduce diabetes mellitus symptoms (Clare-Salzler et al., 1992; Tarbell et al., 2004).

## CONCLUDING REMARKS

The frequency and phenotype of blood mDCs may provide a diagnostic tool for assessing patient risk for inflammatory disease. To this should be added an assessment of the transcriptome and microbiome of blood mDCs. Differences among subjects in their mDC responsiveness to antigen challenge and the co-stimulatory, co-inhibitory phenotype of said mDCs may be particularly amenable to the personalized medicine paradigm. This can lead to early diagnosis before the most extreme of symptoms are manifest and thus improving the efficacy of treatment. The ability of DCs to shape T cell immunity also makes them very attractive targets for clinical therapies targeting cancer, autoimmune disease, and chronic infections. Specifically, subsets of DCs that can drive antigen-specific tolerance are highly desirable for clinical vaccination and prevention of autoimmunity. This antigen-specific tolerance has potential to deliver very productive and specific responses while limiting collateral damage from immune activation.

## Conflict of Interest Statement

The authors declare that the research was conducted in the absence of any commercial or financial relationships that could be construed as a potential conflict of interest.
